# Frequency of central sensitization and nociplastic pain in patients with plantar fasciitis: Central sensitization and nociplastic pain in plantar fasciitis

**DOI:** 10.1007/s00264-025-06462-y

**Published:** 2025-02-27

**Authors:** Gülseren Demir Karakılıç, Melek Melek Aykut Selçuk, Erhan Arif Öztürk

**Affiliations:** 1 Bozok Universitesi, Yozgat, Turkey; 2 Ankara Etlik City Hospital, Ankara, Turkey

**Keywords:** Plantar fasciitis, Nociplastic pain, Central sensitization, Pain-DETECT, Central sensitization scale, Foot function index

## Abstract

**Purpose:**

If the pain persists for a long time in the treatment of plantar fasciitis (PF) or if there is no response to treatment, central sensitization (CS) may develop and the pain may transform into nociplastic pain (NP). This study aimed to evaluate the frequency of CS and NP in patients with PF.

**Methods:**

This cross-sectional study was undertaken between November 2023 and March 2024. The Foot Function Index (FFI) scale, which evaluates the foot’s functionality, was applied to the patient group. The Visual Analog Scale (VAS), which evaluates pain intensity; the Pain-DETECT scale, which evaluates NP; and the Central Sensitization Scale (CSI), which evaluates CS, were applied to patient and control groups.

**Results:**

A total of 206 people were included in the study; 106 were in the patient group with PF, and 100 constituted the control group. While we detected NP in 67 (63.2%) patients according to Pain-DETECT and CS was detected in 91 (85.8%) patients according to CSI among 106 patients with chronic PF; we detected NP in seven (7%) patients according to Pain-DETECT and CS in 44 (44.0%) patients according to CSI among 100 control patients. VAS-score and FFI-pain are moderately and positively correlated with pain-DETECT scores and fairly and positively correlated with CSI scores in the PF group. The pain-DETECT score is moderately and positively correlated with the CSI score in the two groups.

**Conclusions:**

This is the first study to evaluate the presence of CS and NP in PF patients. We found NP and CS to be common in patients with chronic PF. Effective pain management in patients with PF before it becomes chronic can prevent the development of CS and NP.

## Introduction

Plantar fasciitis (PF) is a degenerative syndrome that occurs due to repetitive trauma at the attachment point of the plantar fascia to the calcaneus [1]. PF is one of the most common causes of heel pain, accounting for approximately 80% of cases [2]. Advanced age, pes planus, increased pronation, inappropriate shoe use, obesity, and decreased ankle dorsiflexion are among the detectable causes of the aetiology of PF [3]. Diagnosis is usually based on clinical history and findings of local tenderness.

There are many applied methods in the treatment of PF, such as anti-inflammatory agents, orthostatic supports, night splints, physical therapy methods, corticosteroid applications, night splint use, plastering, rest, lifestyle modification, laser, taping, exercise and Extracorporeal Shock Wave Therapy (ESWT) [4]. Although there are different treatment options for PF, the effectiveness of the treatment often does not last long, and pain and clinical complaints return after a while in some patients [5]. It comes to mind that it turns from nociceptive pain to nociplastic pain (NP), and central sensitization (CS) develops when the pain in PF continues for a long time in this case.

Pain experienced in the musculoskeletal system can be classified according to the underlying pain mechanisms as nociceptive, neuropathic, and nociplastic [6]. Neuropathic pain (NeuP) is pain that occurs as a result of a lesion or disease in the somatosensory nervous system, according to the definition by the International Association for the Study of Pain (IASP) [7]. Nociplastic pain, on the other hand, is pain resulting from altered nociception without tissue or somatosensory damage that causes peripheral nociceptor activation [8]. CS does not confirm NeuP, it points more to nociplastic pain mechanisms. This was supported by consensus study highlighted the features that are common to both neuropathic and nociplastic pain mechanisms [6]. Fibromyalgia, complex regional pain syndrome type 1, chronic low back pain, irritable bowel syndrome, and phantom pain can be given as examples. Clinical criteria for nociplastic pain affecting the musculoskeletal system include no evidence of nociception or NeuP presence [8]. The persistence of heel pain upon waking up in the morning or after sitting for a long time and the persistence of heel tenderness on examination are in favor of nociceptive pain in patients with PF.

Although the primary goal in nociplastic pain treatment is to modify the disease, this is often not possible, and symptomatic pain treatment is usually performed [9]. Therefore, the development of nociplastic pain should be prevented by applying timely and effective treatment for nociceptive pain. There are no prospective case-control studies on nociplastic pain in plantar fasciitis, only a review reported that nociplastic pain may develop in patients with chronic plantar heel pain [10]. NeuP has been studied more in plantar fasciitis. The prevalence of NeuP was reported to be 69%, 28% and 60% in people with PF [11–13], assessed using a range of outcomes including the Patient-Reported Outcomes Measurement Information System (PROMIS) Neuropathic Pain Quality, the Pain-DETECT and the Leeds Assessment of Neuropathic Symptom and Sign (LANSS). No scale that evaluates foot pain and function, such as FFI, was used, and no control patients were included in any of these studies. Therefore, we considered evaluating nociceptive and NP in chronic PF.

CS is the hyperexcitability of neurons in the central nervous system to environmental stimuli. The hyperexcitability of the membrane increases; at the same time, pre-synaptic and post-synaptic activity increases, and CS occurs as the inhibition of dorsal horn neurons decreases in response to environmental stimuli. It was reported that psychological variables play a role in plantar heel pain and that CS should be taken into consideration in a review [10]. Sensorimotor examination was performed in long-distance runners with medial plantar pain; it was reported that CS caused the pain [14] and CSI scores were found to be 40 and above in 25% of patients with PF [15].

As risk factors such as obesity, prolonged standing, or sedentary lifestyle persist in patients with PF, the condition causing degeneration and become chronic [16]. Despite conservative treatment in chronic PF, patients’ complaints such as pain, numbness, burning, and cramps may continue. In such a case, if we can distinguish whether the pain is nociceptive or nociplastic, we can treat NP if it is present, instead of trying different conservative treatments that have unnecessary additional costs [10]. On the other hand, if we know the frequency of NP in patients with chronic PF; we can reduce this frequency if we prevent chronic degeneration with lifestyle changes after conservative treatment in patients with acute PF [17]. No study has evaluated CS and NP together in patients with PF. We aimed to evaluate the frequency of central sensitivity and NP and in PF patients with heel pain for at least six months, compared to a control patient of similar age and gender, with chronic musculoskeletal pain, without foot pain in this study.

## Patient and methods

### Study design

This cross-sectional study was undertaken between November 2023 and March 2024. Written and verbal informed consent was obtained from the patient and control group participants. The study protocol was approved by the Human Research Ethics Committee (AEŞH-EK 1-2023-612). Patient enrollment start date: 10/19/2023, and ClinicalTrials.gov registration ID: NCT06348017.

### Patients

The study was commenced after obtaining the approval of the local ethics committee and the informed consent of the participants. Patients who applied to the City Hospital Physical Medicine and Rehabilitation outpatient clinic with heel pain for six months or longer, between the ages of 18–65, who have heel pain when getting out of bed in the morning or after long periods of inactivity, who have localized tenderness on the medial side of the calcaneal tuberosity upon palpation were examined by two Physical Medicine and Rehabilitation specialists who had at least eight years of specialized experience; and patients diagnosed with PF were consecutively invited to participate in the study; patients who agreed to fill out the questionnaires and sign written and verbal informed consent were included in the study, as a patient group. After completing the surveys of patients with PF, the gender and age distribution of the patient group was determined. We chose patients with chronic musculoskeletal pain as the control group instead of healthy controls since VAS and the Pain-DETECT scale included questions about pain. Patients who applied to the City Hospital Physical Medicine and Rehabilitation outpatient clinic with any musculoskeletal pain for at least six months, without foot pain, and of similar age and gender were included in the study as the control group. Patients with a history of diabetes, hypothyroidism, and congestive heart failure, a history of malignancy, with vasculitis, neurological diseases that may cause NP, lumbar discopathy, those with a previous history of fracture or surgery in the heel area, received injections or ESWT treatment due to heel pain in the last three months, rheumatic diseases that may affect pain such as rheumatoid arthritis, ankylosing spondylitis, and severe circulatory disorders on the side with pain, were excluded from the study.

All patients’ personal information (age, gender, occupation, education information), general health information (smoking and alcohol use information, known chronic disease history, body mass index), severity of heel pain, and when it started were recorded. Patients were screened for additional diseases such as fibromyalgia, restless legs syndrome, anxiety, and depression, both through the questioning in the second part of the CSI questionnaire and from the electronic patient card, where the patients’ past diagnoses can be seen. All patients with PF included in the study had radiographs taken, and the radiographs were reviewed by the same two Physical Medicine and Rehabilitation specialists who completed the questionnaires. The Foot Function Index (FFI) scale, which evaluates the functionality of the foot, was applied to the patient group; the Visual Analog Scale (VAS), which evaluates pain intensity; the Pain-DETECT scale, which evaluates NP, and the CSI, which evaluates CS, were applied to the patient and control groups. The details of the implementation of the instruments are as follows:

#### Visual analog scale (VAS)

For the Evaluation of Pain. For this evaluation, the patient is asked to mark their pain and fatigue severity on a horizontal 10-cm line with the number 0 on one end representing “no pain or no fatigue” and the number 10 on the other end indicating “very severe pain or very severe fatigue.”

#### Foot function index (FFI)

Sub-parameters of FFI: pain, disability, and activity limitation. It is a scale consisting of a total of 23 items. The pain subscale includes nine items and measures the severity of foot pain in different situations. The disability subscale consists of nine items and evaluates the severity of the person’s difficulty performing functional activities due to foot problems. The activity limitation subscale includes five items and measures the person’s activity limitations due to foot problems. People answer the questions using the Visual Analogue Scale (VAS), considering their foot conditions in the previous week. A higher score indicates more pain, disability, and activity limitation. Turkish validity and reliability study was conducted by Yalıman et al. [18] by modifying and adding minor explanations in the information part and for questions 7, 8, and 9 for pain, 3, and 8 for disability, and 2, 4, and 5 for activity limitation to make the Foot Function Index appropriate for use in Turkish speaking patients [18].

#### Pain-DETECT

The survey consists of nine questions, with a score range of 1–38. Scores of 12 and below are considered as ‘NP component is unlikely,’ scores between 13 and 18 are regarded as ‘NP component is uncertain,’ and scores of 19 and above are considered as ‘NP component is possible.’ Turkish validity and reliability study was conducted by Alkan et al. in 2013 [19]. This study’s sensitivity and specificity were 90% and 67.5%, respectively, for the cutoff value ≤ 12. Scores ≤ 12 represented a negative predictive value as 87%. The Pain-DETECT, which is poor at ruling in NeuP can detect NP [20].

#### Central sensitization scale (CSI)

The survey consists of 25 questions. Each question is scored between 0 and 4 (0 = never, 4 = always). A score of 40 and above 31 indicates the presence of CS with 81% sensitivity and 75% specificity. CS severity is defined as 0–29 subclinical, 30–39 mild, 40–49 moderate, 50–59 severe, and 60 or above very severe. Turkish validity and reliability study was conducted by Keleş et al. in 2021 [21]. The internal consistency (Cronbach’s alpha) was 0.92, and the intraclass correlation coefficient was 0.93 in this study.

### Statistical analysis

Sample analysis was performed using the Openepi application https://www.openepi.com/SampleSize/SSPropor.htm). Based on the study by Wheeler et al. (Wheeler P. C. (2017). in which 29% of 126 patients diagnosed with PF had possible NP, it was found that 91 patients should be included in the study with a 95% confidence interval for this study. Data were analyzed using the Statistical Package for Social Sciences, version 25.0 (SPSS Inc., Armonk, NY). The normality of the numerical data distribution was examined using the Kolmogorov-Smirnov test. Continuous variables with normal distribution were presented as mean and standard deviation, and those without normal distribution were given as median and interquartile range (IQR; 25th -75th percentiles). The categorical variables were expressed as frequency and percentages. The categorical data were compared using the Chi-squares test, according to the frequency of expected counts, Pearson Chi-square, Fischer exact, and Yate’s continuity correction tests were applied. The numerical variables with or without parametric distribution were compared with the independent samples t-test and Mann-Whitney U, respectively. Spearman correlation analysis was performed to determine variables associated with VAS score, foot function index-pain, pain-DETECT score, and CSI scores in plantar fasciitis group. Correlation efficiency of < 0.3, 0.3–0.6, 0.6–0.8 and ≥ 0.8 were accepted as low, fair, moderate and strong correlations, respectively. (Chan Y. H. (2003). Biostatistics 104: correlational analysis. *Singapore medical journal*, *44*(12), 614–619.): The confidence interval was 95%, and the accepted margin of error was 5%. A value of *P* < 0.05 was considered statistically significant.

## Results

A total of 206 people were included in the study, 106 of them as a patient group with PF; 100 were determined as the control group. Demographic features of the participants are given in Table 1. No significant difference was found between the groups regarding age, gender, BMI, education, occupation, and smoking status. The ratio of married is higher in the patient group, and therefore, the ratio of single is higher in the control group (*p* < 0.001).

**Table 1:** Demographic features of the participants (*n* = 206)

	PF group (*n* = 106)	Control group (*n* = 100)	*p*
	*N*/% or median (IQR)		
Age	53.0 (12.0)	51.0 (16.0)	0.616^a^
Gender			
Female	87(82.1)	83 (83.0)	
Male	19 (17.9)	17(17)	
BMI	29.8 (4.9)	29.3 (5.7)	0.856^a^
Education			0.924^b^
Illiterate	9 (8.5)	11 (11.0)	
Primary school	55 (51.9)	54 (54.0)	
Secondary school	11 (10.4)	10 (10.0)	
Highschool	22 (20.8)	19 (19.0)	
University	9 (78.5)	6 (6.0)	
Occupation			.0613^b^
Unemployed	84 (79.2)	84 (84.0)	
Worker	8 (7.5)	7 (7.0)	
Officer	14 (13.2)	9 (9.0)	
Marital status			**< 0.001** ^**b**^
Married	103(97.2)	76 (76.0)	
Single	2 (1.9)	22 (22.0)	
Widow	1 (0.9)	2 (2.0)	
Smoking	19 (17.9)	13 (13.0)	0.344^c^

PF: plantar fasciitis, N: number, BMI: Body mass index, ^a^Mann-Whitney U test, ^b^Pearson-chi square test, ^c^Fischer exact test

The frequency of the presence of systemic disease, restless leg syndrome, chronic fatigue syndrome, Fibromyalgia syndrome, migraine or tension-type headache, anxiety disorder, and depression is significantly higher in the PF group (*p* < 0.05). Because of the low number of patients with temporomandibular disorder, whiplash injury, and multiple chemical sensitivity, no comparison could be made between the groups. According to the pain-DETECT categorization, the rate of NP is higher in the patient group, while the groups were similar in terms of uncertain category; also, the pain-DETECT score is significantly higher in the PF group (*p* < 0.001). CSI score is higher in the PF group than in the control group, so the ratio of CS is higher in the PF group (*p* < 0.001). The clinical characteristics of the PF group and the comparison of clinical variables between the groups are presented in Tables 2 and 3, respectively.

**Table 2:** Clinical characteristics of the plantar fasciitis group (*n* = 106)

	*N*/%	Median (IQR)
Symptomatic side		
Right	25 (23.58)	
Left	9 (8.49)	
Bilateral	72 (67.92)	
Pain duration (months)		24.0 (29.0)
Treatments before		
NSAIDs intake	68 (64.2)	
Plantar fascia steroid injection	50 (47.2)	
ESWT	54 (50.9)	
Neuropathic treatment agents	35 (33.0)	
VAS score (0-100)		
Right foot		80.0 (20.0)
Left foot		75.0 (20.0)
FFI total score		
Right		61.5 (24.0)
Left		57.0 (50.0)
FFI-pain score		
Right		64.0 (32.0)
Left		56.0 (60.0)
FFI-disability		
Right		70.0 (22.0)
Left		67.0 (82.0)
FFI-activity		
Right		36.0 (21.0)
Left		36.0 (48.0)

N: number IQR: Interquartile range, NSAIDs: Non-steroidal anti-inflammatory drugs, ESWT: Extracorporeal Shock Wave Therapy, VAS: Visual analog scale, FFI: Foot function index

**Table 3:** Comparison of clinical variables between the groups

	PF group (*n* = 106)	Control group (*n* = 100)	*p*
	*N*/% or median (IQR)		
Area(s) of pain (n/%)		Shoulder pain 40(%40)	
		Lateral elbow pain 30(%30)	
		Knee pain 30(%30)	
Diagnoses of chronic pain (n/%)		Rotator Cuff Syndrome 40(%40)	
		Lateral elbow tendinopathy 30(%30)	
		Patellar tendinopathy 30(%30)	
Pain duration (months) (**median (IQR))**	24.0 (29.0)		
Past treatments (n/%)		NSAIDs intake 80(%80)	
		Steroid injection 48 (%48)	
		Physiotherapy 50 (%50)	
VAS score	80. 0 (20.0)		
Systemic disease (n/%)	44 (31.5)	26 (26.0)	**< 0.01** ^**a**^
Restless leg syndrome	44 (41.5)	6 (6.0)	**< 0.001** ^**a**^
Chronic fatigue syndrome	35 (32.1)	6 (6.0)	**< 0.001** ^**a**^
Fibromyalgia syndrome	46 (43.4)	5 (5.0)	**< 0.001** ^**a**^
Temporomandibular disorder	5 (4.7)	1 (1.0)	
Migraine or tension-type headache	56 (52.8)	34 (34.0)	**< 0.001** ^**a**^
Irritable bowel syndrome	13 (12.3)	1 (1.0)	**< 0.01** ^**a**^
Multiple chemical sensitivity	1 (0.9)	-	
Whiplash injury	2 (1.9)	1 (1.0)	
Anxiety disorder	46 (43.4)	24 (24.0)	**< 0.01** ^**a**^
Depression	28 (26.4)	13 (13.0)	**0.023** ^**a**^
Pain-DETECT score (**median (IQR))**	22.0 (13.0)	6.0 (10.0)	**< 0.001** ^**b**^
Pain-DETECT group (**median (IQR))**			**< 0.001** ^**a**^
Negative	17 (16.0))	79 (79.0)	
Positive	67 (63.2)	7 (7.0)	
Unclear	22 (20.8)	14 (14.0)	
CSI-central sensitization (score of ≥ 40)	91 (85.8)	44 (44.0)	**< 0.001** ^**a**^
CSI score (**median (IQR))**	57.0 (26.0)	38.0 (15.0)	**< 0.001** ^**b**^

PF: plantar fasciitis, N: number, CSI: Central sensitization inventory, ^a^Fischer exact test, ^b^Mann-Whitney U test, CSI: Central sensitization inventory

VAS-score is positively and moderately correlated with FFI-pain (Rho = 0.618, *p* < 0.001), positively and fairly correlated with pain-DETECT score (Rho = 0.618, *p* < 0.001), also poorly and positively correlated with CSI score (Rho = 0.225, *p* = 0.020). FFI-pain is moderately and positively correlated with pain-DETECT scores (Rho = 0.662, *p* < 0.001) and also fairly and positively correlated with CSI score (Rho = 0.503, *p* < 0.001). Pain-DETECT score is moderately and positively correlated with the CSI score (Rho = 0.667, *p* < 0.001) (Table 4).

**Table 4:** Spearman correlation analysis of VAS score with foot function index-pain, pain-DETECT score, and CSI scores in plantar fasciitis group

Spearman’s rho		VAS score	FFI-pain	Pain-DETECT score	CSI score
VAS score	Rho	1.000	0.618**	0.365**	0.225*
	*P*	.	**< 0.001**	**< 0.001**	**0.020**
FFI-pain	Rho	0.618**	1.000	0.662**	0.503**
	*P*	**< 0.001**	.	**< 0.001**	**< 0.001**
Pain-DETECT score	Rho	0.365**	0.662**	1.000	0.667**
	*P*	**< 0.001**	**< 0.001**	.	**< 0.001**
CSI score	Rho	0.225*	0.503**	0.667**	1.000
	*P*	0.020	**< 0.001**	**< 0.001**	.

VAS: Visual analog scale, FFI: Foot function index, CSI: Central sensitization inventory

** Correlation is significant at the 0.01 level (2-tailed). * Correlation is significant at the 0.05 level (2-tailed)

## Discussion

While we detected NP in 67 (63.2%) patients according to Pain-DETECT and CS was detected in 91 (85.8%) patients according to CSI among 106 patients with chronic PF; we detected NP in seven (7%) patients according to Pain-DETECT and CS in 44 (44.0%) patients according to CSI among 100 control patients. VAS-score and FFI-pain are moderately and positively correlated with pain-DETECT scores and fairly and positively correlated with CSI scores in the PF group. The pain-DETECT score is moderately and positively correlated with the CSI score in the two groups.

PF was more common in women (82.1%) in PF group, similar to the literature, and the average age of the patients was 53.0 ± 12.0 also similar to the literature [2, 22, 23]. Most of the patients with PF were overweight, and their average BMI was 29.8 ± 4.9, similar to the literature [22, 24]. Most patients with PF were not working similarly to the literature [2, 22]. Since the average age of the patients in the patient and control groups was 52.31 and 82.5% were women, the unemployment rate in our study was 81.6%, consistent with age and gender in our country [25]. Most of the patients with PF were bilateral (67.92%), similar to the literature [22, 26].

NeuP has been studied more than NP in different tendinopathies in the literature. The prevalence of NeuP was reported to be 10.9% in people with rotator cuff tears according to Pain-DETECT [27]; 15.8% in people with rotator cuff tears according to Douleur Neuropathic 4 questionnaire (DN4) [28]; 31% in people with greater trochanteric pain syndrome, 10% in people with patellar tendinopathy, 26% in people with mid-substance Achilles tendinopathy, 28% in people with insertional Achilles tendinopathy according to Pain-DETECT [12]; 58.11% in people with carpal tunnel syndrome according to The Leeds Assessment of Neuropathic Symptom and Sign (LANSS) [29]; 49% in people with lateral elbow tendinopathy, 33% in people with greater trochanteric pain syndrome, 36% in people with patellar tendinopathy, 48% in people with non-implanting Achilles tendinopathy, 49% in people with established Achilles tendinopathy according to LANSS [13]; and 29.5% in people with lateral epicondylitis according to Pain-DETECT [30]. We found NP in 63.2% in patients with chronic PF in our study according to Pain-DETECT. We found NP more frequently in chronic PF due to characteristics such as the plantar fascia being quite wide and close to the tibial nerve branches; the high rate of women and obesity; and the median duration of symptoms being 24 months.

Plantar fascia was examined in terms of NP in two studies in the literature. The tibial nerve branches of 41 patients with persistent heel pain were examined in a study conducted in Spain; it has been reported that if VAS is high, this may cause NP since the neural structures are attached to the plantar fascia, even though there is no compression on the nerve in the MRI [31]. A 56-year-old female patient with bilateral PF resistant to conservative treatment was diagnosed with Baxter neuropathy caused by compression of the inferior calcaneal nerve, as atrophy of the abductor digiti minimi muscle was shown on MRI in a case report, and the nerve was surgically released bilaterally. It was reported that NP may develop in patients with refractory PF due to the proximity of the plantar fascia and calcaneal nerve in the case report [32]. Since the average symptom duration in our study was 24 months, long-lasting irritation could stimulate the nerves close to the plantar fascia, which triggers NP [17].

There are no prospective case-control studies on nociplastic pain in plantar fasciitis, NeuP in PF has been investigated in three studies, one of which is an oral report and two prospective cohort studies without a control group in the literature. The prevalence of NeuP was reported to be 69%, 28% and 60% in people with PF [11–13], assessed using a range of outcomes including the Patient-Reported Outcomes Measurement Information System (PROMIS) Neuropathic Pain Quality, the Pain-DETECT and the Leeds Assessment of Neuropathic Symptom and Sign (LANSS). We found NP in 63.2% in patients with chronic PF in our study according to Pain-DETECT. The rate of women was high, and the average age was 53 in our study, similar to these studies. Our study had a control group, and we found that NP correlated with FFI, a scale that evaluates the foot’s function, unlike in these studies. Pain-DETECT questionnaire NeuP is a scale with high sensitivity and low specificity and can detect nociplastic pain [20, 33]. Therefore, we used Pain-DETECT to detect nociplastic pain.

CS has been studied in different tendinopathies in the literature. One review reported that CS was less common in lower extremity tendinopathies than in upper extremity tendinopathies [34]. However, only one study on lower extremity tendinopathies was referenced in the review; 19 patients with patellar tendinopathy and 30 patients with Achilles tendinopathy were included in the study, and no difference was found in terms of CS with the control group [35]. The prevalence of CS was reported to be 29% in patients with trochanteric pain syndrome, 17% in patients with patellar tendinopathy, 20% in patients with mid-substance Achilles tendinopathy, 22% in patients with insertional Achilles tendinopathy [15]; 44% in patients with greater trochanteric pain syndrome [36]; 60% in patients with carpal tunnel syndrome [37]; and ranged from 29 to 77% in patients with shoulder pain in a meta-analysis [38]; according to CSI. There is only one study investigating the frequency of CS in PF. CS was found in 27% in patients with PF according to CSI [15]. We found CS in 85.8% in patients with chronic PF, according to CSI in our study. The reason why we found CS more frequently in chronic PF may be due to plantar fascia-related features, the average age of the patients in the study being 52, the high rate of women and obesity, and the average symptom duration being 24 months.

There was no blinded assessor because the assessors knew whether the patients had plantar fasciitis or were controls. The questionnaires used were not sufficient to diagnose CS or NP, so results must be interpreted cautiously. CSI on its own is not enough to diagnose CS. Future work needs to combine this with Quantitative Sensory Testing to validate the findings in the current study. Another limitation was the lack of assessment of somatic symptoms associated with NP (e.g., fatigue, sleep disturbances, widespread pain, mood, and memory difficulties). The last limitation was that the effect of NP and CS on treatment and prognosis in PF was not evaluated.

## Conclusions

This study is the first in the literature to evaluate the presence of CS and NP in PF patients. We found CS and NP to be common in patients with chronic PF. We found that as the pain intensity of patients with PF increased, the frequency of CS and PF increased as the function was impaired, according to FFI and VAS. If we effectively treat pain in patients with PF before it becomes chronic, we can prevent the development of CS and NP. Additionally, we can distinguish whether the pain is nociceptive or nociplastic; we can treat NP if it is present instead of trying different conservative treatments that have unnecessary additional costs.

## Data Availability

All the data and materials are available upon requests from the corresponding author.
